# Suicide risk assessment and management needs of pre-examination and triage nurses in emergency departments: A qualitative study

**DOI:** 10.12669/pjms.41.4.10501

**Published:** 2025-04

**Authors:** Chang Du, Dan Shen, Jing Yuan, Ruihuai Zhang, Cong Fu, Xiaoping Yin

**Affiliations:** 1 Chang Du, School of Nursing, Hebei University, Baoding 071000, Hebei, China; 2 Dan Shen, Department of Urology, Affiliated Hospital of Hebei University, Baoding 071000, Hebei, China; 3 Jing Yuan, School of Nursing, Hebei University, Baoding 071000, Hebei, China; 4 Ruihuan Zhang, Department of Gastroenterology, Affiliated Hospital of Hebei University, Baoding 071000, Hebei, China. School of Nursing, Hebei University, Baoding 071000, Hebei, China; 5 Cong Fu, Department of Nursing, Affiliated Hospital of Hebei University, Baoding 071000, Hebei, China. School of Nursing, Hebei University, Baoding 071000, Hebei, China; 6 Xiaoping Yin Affiliated Hospital of Hebei University, Baoding 071000, Hebei, China. School of Nursing, Hebei University, Baoding 071000, Hebei, China

**Keywords:** Emergency service, Qualitative Research, Suicide, Triage

## Abstract

**Objective:**

To explore the barriers and management needs of pre-examination and triage nurses in emergency departments in assessing suicide risk of patients.

**Methods:**

Using descriptive phenomenology, this study performed semi-structured in-depth interviews with nurses in emergency departments of two third grade Class-A hospitals in Baoding City from September 10 to October 1, 2023. The thematic analysis of phenomenological data was conducted using Colaizzi’s seven steps analysis method.

**Results:**

According to the interview with 14 nurses, this study identified three themes and nine sub-themes, including identifying suicide risk characteristics of patients (suicide and self-injurious behaviors, psychological and mental state, medical history and life experience), barriers in assessing suicide risk of patients (suicide stigmatization, uncertainty of responsibility, and occupational psychological stress of nurses), and suicide risk management needs (concise and effective suicide risk assessment process, knowledge and skills training on suicide prevention, and organizational support).

**Conclusion:**

The suicide risk assessment of patients in emergency pre-examination and triage should be further optimized from the aspects of organizational management and clinical practice, such as improving resource allocation, establishing emergency suicide risk assessment workflow, and developing training programs to improve the overall suicide prevention literacy and management ability of pre-examination and triage nurses in emergency departments.

## INTRODUCTION

Suicide is a major public health issue worldwide. The World Health Organization (WHO) reports that approximately 700,000 people die from suicide each year. According to prior research, the contact rate with primary health care was 80% in the year prior to suicide, and 44% at one month.^1^ Among all patients in emergency settings, the proportion of suicidal ideation was as high as 11%, while the detection rate was only 3%.^2^ Emergency pre-examination and triage refer to a rapid evaluation of emergency patients, the prioritization and triage of patients based on their medical conditions and severity.^3^ It is the first critical step to ensure correct treatment for patients in the emergency department, which directly affects the therapeutic effect as well as the efficiency of medical and nursing services.

Screening for suicide patients is one of the important functions of emergency pre-examination and triage.^4^ Suicide risk management system in foreign countries has become mature for patients admitted to the emergency department. However, so far, there is still no guideline or consensus in China for evaluating the suicide risk of patients in emergency departments; moreover, professional and technical levels of medical staff for triage varied in various hospitals, resulting in low quality of triage.^5^ Accordingly, this study was conducted to explore the process and needs of emergency pre-examination and triage nurses in assessing patient suicide risk using descriptive phenomenological analysis, so as to provide reference for suicide risk management and training of pre-examination and triage nurses in emergency departments.

## METHODS

This was a descriptive study. The interviewees were nurses and nurse managers in emergency departments of two third-grade class-A hospitals in Hebei Province from September 10 to October 1, 2023. For sampling, a notice of voluntary participation in the study was issued by the head nurse.

### Ethics approval

The study was approved by the Institutional Ethics Committee of Affiliated Hospital of Hebei University (No: HDFYLL-KY-2023-077; Date: September 25, 2023), and written informed consent was obtained from all participants.

### Inclusion criteria:

- Nurses engaged in pre-examination and triage for ≥1 year(s).
- Nurses with normal language competence and thinking ability.
- Nurses who agreed to participate in the interview and provided written informed consent.

### Exclusion criteria:

- Nurses undergoing standardized training and student nurses.
- Nurses in the emergency department who were absent due to sick leave, maternity leave, continuing education, etc.

Considering factors such as gender, age, years of service, professional title and position, maximum difference sampling was adopted for screening eligible subjects from the list of applicants. The sample size was determined when the themes reached saturation, with two more nurses interviewed before the end of sampling until no new information was presented. This study included 14 nurses in emergency departments eventually, with the general data shown in Table-I.

**Table-I:** General data of the subjects of study.

No.	Age (years)	Gender	Marital status	Positional title	Education Level	Years of service	Years of experience in pre-examination and triage
N1	36	Female	Married	Nurse-in-charge	Bachelor	14	10
N2	42	Female	Married	Nurse-in-charge	Bachelor	20	15
N3	30	Female	Unmarried	Nurse-in-charge	Bachelor	7	2
N4	29	Male	Married	Primary nurse	Associate degree	6	1
N5	44	Male	Married	Nurse-in-charge	Bachelor	21	15
N6	45	Female	Married	Associate chief nurse	Bachelor	24	10
N7	37	Female	Married	Associate chief nurse	Master	15	10
N8	29	Male	Unmarried	Primary nurse	Associate degree	7	1
N9	32	Male	Unmarried	Nurse-in-charge	Bachelor	10	4
N10	35	Female	Married	Nurse-in-charge	Bachelor	10	5
N11	31	Female	Unmarried	Primary nurse	Bachelor	8	5
N12	33	Female	Married	Nurse-in-charge	Bachelor	9	5
N13	30	Male	Married	Primary nurse	Bachelor	6	1
N14	34	Male	Married	Nurse-in-charge	Bachelor	11	8

### Data collection:

A semi-structured interview was conducted for data collection.The interview outline was formulated preliminarily according to literature review, which was further revised to establish the official version by pre-interviewing two nurses in emergency departments before the formal interview. The final interview outline was:

1) How did you identify a patient’s suicide risk during triage?
2) What would you think and do if the patient was determined to have a risk of suicide?
3) What factors can effectively facilitate your assessment of a patient’s suicide risk during triage?
4) What were the barriers that interfered with effective assessment of patient suicide risk during triage?
5) What suggestions do you have for effectively assessing a patient’s suicide risk during triage?
6) What training needs for knowledge, skills and other aspects do you have for assessing patient suicide risk?

To ensure interview quality, interviewers were clinical nurses with work experience in emergency departments. Prior to the interview, the interviewees were informed of and provided the written informed consent. After gaining the trust and obtaining the consent of the interviewee, on-site live recording and note were performed throughout the entire process, with the time and site of the interview determined jointly. The interview lasted for about 30-60 minutes. During the interview, the interviewer flexibly adjusted the interview sequence, listened attentively, asked appropriate questions, and recorded nonverbal information such as the interviewee’s facial expressions, gestures, and emotions.

### Data analysis:

The recorded content was transcribed into text word for word within 24 hafter the interview, with the nonverbal actions of the interviewee annotated in the text. Data verification was completed by another member of the research group. Each interviewee’s text data was analyzed using Colaizzi’s 7-step method^6^ for theme extraction. The specific steps were as follows:

1) Be fully familiar with and understand all content through repeated review of the materials by the researcher
2) Identify meaningful statements
3) Establish the significance
4) Cluster themes
5) Describe comprehensively
6) Generate basic structure
7) Verify basic structure.

In case of any disagreement on the extracted theme, the research group would discuss jointly to determine the theme finally.

## RESULTS

### Suicide and self-injurious behaviors:

During pre-examination and triage, nurses can make preliminary judgments and evaluations based on the hidden information in language communication. N8: “Some patients were admitted to the hospital with injuries at the wrist with obvious neat cuts, which were suspected to be suicidal and self-injurious behaviors”.

Some experienced nurses believed that suicidal intentions were difficult to be found in some patients who showed indifference, few words, and lack of initiative, which would exhibit a risk of implicit suicide. N2: “Some patients were silent, indifferent, in low spirits and depressed. Such type of patients with melancholy feature would run out when they were not being noticed”.

### Psychological and mental state:

The safety of patients with abnormal psychological and mental states might be highly concerned by nurses in the emergency department to evaluate their risk of suicide. N12: “Some patients might be in a state of staring blankly, at a loss what to do, or confusion in answering questions. We might preliminarily determine that the patient had some psychological disorders, and could only employ empirical approaches since it was beyond our ability to make a clear diagnosis”.

*Medical history and life experience*. During triage, nurses would check the medical records or chief complaints of patients to confirm their medical history (schizophrenia, depression, anxiety, etc.), and whether they had recently experienced negative life events such as family member death, job loss, divorce, etc. N6: “We were sensitive to patients’ medical history of mental illness when checking their medical records”.

### Suicide stigmatization:

During triage, the vast majority of nurses were unwilling to publicly inquire and discuss suicide issues when identifying patients’ suicide risk, and hold a taboo attitude towards “death”, “suicide”, “reasons for suicide”, etc. N1: “No one would directly ask patients if they had suicidal tendencies, but inquired their reasons for discomfort to let patients express themselves”.

Some nurses held negative emotions and stereotypes towards patients who committed suicide through taking poison, jumping off buildings, cutting their wrists, etc. N6: “Suicide patients were frightening and it was unsure when they would have another accident, just like a time bomb”. N8: “Some young people chose to take poison and jump off buildings because of arguments with their families. Many people were struggling to live, but they did not value life at all”.

### Uncertainty of responsibility:

Most nurses in emergency departments believed that they were the most suitable candidates for assessing patient suicide risk. It was speculated that compared to other medical staff, nurses had the longest duration of contact with patients and the most opportunities for communication, which was consistent with the results of a foreign study.^4^ While some others thought that the safety of suicide patients should be ensured primarily by their families. N1: “Psychologists should be responsible for intervening patients who committed suicide considering their professionalism in diagnosing mental illnesses, applying psychological interventions, and prescribing authority”. N11: “For patients at risk of suicide, we would repeatedly emphasize to their families that they must keep close to and take good care of the patients”.

### Occupational psychological stress of nurses:

Some nurses might experience heavy psychological stress during medical practice in the environment, producing a negative impact on the management of patients at risk of suicide. N7: “We worked in the emergency department with heavy workload and tight work schedule in a state of mental tension. Sometimes it was difficult for us to evaluate or comfort a patient who had a desire to commit suicide”. N6: “The emergency department prioritized patients with life-threatening conditions definitely. We felt like there was not enough time to evaluate the suicide risk of patients”.

### Concise and effective suicide risk assessment process:

As indicated by the nurses interviewed in our study, there was a need for concise and effective suicide risk assessment process as standardized process would improve triage efficiency. N1: “It was necessary to assess the risk of suicide for each patient. Young nurses were inexperienced generally, and standardized processes might offer a solution for the proposed problem. However, it should be concise and effective to ensure the efficiency of triage”.

### Knowledge and skills training on suicide prevention:

Most nurses did not receive any training on suicide prevention, but agreed with the necessity of training. Targeted training can improve nurses’ knowledge and skills in suicide prevention. N13: “We did not participate in any training on suicide prevention. The method of communicating with patients was an important issue”. N7: “I intended to learn the method to guide and comfort patients well if there was training”.

### Organizational support:

Most nurses believed that patient suicide prevention of patients in the emergency department relied on the organizational support of the hospital, such as sound systems, human resource allocation, intra- and inter-department solidarity and coordination, and cultural atmosphere for preventing suicide. N4: “Our anxiety would be alleviated if we got support and guidance when we encountered difficulties and reported to the head nurse and director”. N7: “It would disturb and worry me if the next shift did not take this safety issue seriously during shift handover”.

## DISCUSSION

Findings in this study suggest that establishment of suicide risk assessment workflow, training of knowledge and skills on suicide prevention, and organizational support are required by pre-examination and triage nurses in emergency departments. Therefore, it proposes requirements for creating an institutional culture of “no-suicide”, further improving resource allocation, establishing suicide risk assessment workflow, and developing training plans to enhance the overall suicide prevention literacy and management ability of pre-examination and triage nurses in emergency departments.

According to the interview with 14 nurses totally, this study identified three themes and nine sub-themes, including identifying suicide risk characteristics of patients (suicide and self-injurious behaviors, psychological and mental state, medical history and life experience), barriers in assessing suicide risk of patients (suicide stigmatization, uncertainty of responsibility, and occupational psychological stress of nurses), and suicide risk management needs (concise and effective suicide risk assessment process, knowledge and skills training on suicide prevention, and organizational support).

For the first time, this study systematically discussed the obstacles and management needs of suicide risk assessment from the perspective of emergency pre-examination triage nurses, filling the gap of relevant domestic research. Consistent with other research, this study found that emergency nurses face multiple barriers in suicide risk assessment.^7^ Identification and management of suicide risk is complicated that even requires specific knowledge and skills in psychiatry.^8^ However, for this reason, involvement of the minority is insufficient for suicide prevention. According to experts, nurses are key “gatekeepers” for preventing patient suicide by playing multiple roles such as educators, identifiers, evaluators, planners, caregivers, communicators, protectors, referrals, and researchers.^9^

In institutions providing medical services for patients at risk of suicide, principles of scientific nurse-patient ratio and hierarchical-level ratio during nursing human resource allocation should be followed to meet the unpredictable safety needs of patients with significant suicidal intents. Meanwhile, psychiatrists should be 24 hour standby when nurses contact. Sufficient time, space, and tools should be available for nurses to assess suicide risk for observing and monitoring patients to meet their needs. Measurement tools such as Suicide Behaviors Questionnaire, Beck Suicide Intention Scale, and Depression Anxiety Stress Scale have been proven to be simple, reliable, and effective in evaluating patients’ mental health and suicide ideation.^10^-^12^ In addition, future research could explore how suicide risk assessment tools, such as the Beck Suicide Intent Scale, can be integrated into the emergency department pre-screening triage process to improve the accuracy and efficiency of the assessment.

In foreign countries, “gatekeeper” training has been recognized to be the most widely used form of preventive intervention for suicide, which trains a special population to identify individuals at risk of suicide and take interventions.^13^ A research has identified the merit of targeted patient suicide risk training for standardizing nursing behaviors of nurses in the emergency department.^14^ At present, the programs of short-term training and long-term “gatekeeper” training for medical staff to prevent patient suicide, with various learning forms such as knowledge education, group focus interviews, case analysis, etc.^15^ As evidenced in several prior studies^16^-^17^, training on suicide prevention-related knowledge, communication skills, psychological intervention skills, and cultivation of empathy skills might enhance the overall confidence and self-efficacy of nurses in preventing suicide. However, compared with the mature “gatekeeper” training system abroad, this study found that domestic nurses generally lack systematic suicide prevention training, which may be related to the inadequacy of relevant domestic policies and resources.

Multiple theoretical frameworks and practical experiences have been developed in foreign countries, which are good references for preventing patient suicide. For instance, “Caring for Adult Patients with Suicide Risk: A Consensus Guide for Emergency Departments” issued by the Suicide Prevention Resource Center in the United States has proposed comprehensive strategies for risk factor assessment, suicide intervention, referral, and follow-up of attempted suicide patients. A research has showed they established a suicide risk assessment process for suicide patients in emergency departments^18^, requiring medical staff in the emergency department to comprehensively evaluate the suicide protective and risk factors of patients, collect and integrate generated data to determine the risk level, conduct assessment of suicide risk in psychiatry and suicide intervention, etc.In China, it is still in the initial exploration stage, despite gradual development in relevant studies.

Suicide stigmatization is a negative attitude towards suicide or attempted suicide resulted from discrimination and prejudice due to insufficient awareness of suicide.^19^ Similar to this study, a survey of suicide attitudes among 268 medical staff in the emergency department was carried out by a domestic researcher Zeng G et al.^20^ Actually, it has been revealed that public discussion on suicide would not result in negative emotions or lead to suicide, but was necessary. Expression of suicidal thoughts itself, to some extent, offers a critical opportunity for intervention on such patients.^21^ These findings provide important reference for the construction of emergency suicide prevention system in China.

### Limitations:

However, this study also has some shortcomings, such as a small number of samples. In view of this, more samples should be included in future studies to further validate the findings of this study.

## CONCLUSIONS

For pre-examination and triage nurses in emergency departments, suicide risk assessments on patients are generally realized based on patients’ suicidal and self-injurious behaviors, psychological and mental states, medical history, and life experience. However, the efficiency of suicide risk assessment and management by nurses may be compromised owing to the presence of barrier factors such as suicide stigmatization, uncertainty of responsibility, and heavy occupational psychological stress among nurses.

### Authors’ Contributions:

**CD,** **DS** and **XY:** Carried out the studies, participated in collecting data, and drafted the manuscript, and are responsible and accountable for the accuracy or integrity of the work.

**JY, RZ** and **CF:** Literature search, performed the statistical analysis and participated in its design.

All authors have read and approved the final manuscript.
